# A Combination of Leaf Rust Resistance Genes, Including *Lr34* and *Lr46*, Is the Key to the Durable Resistance of the Canadian Wheat Cultivar, Carberry

**DOI:** 10.3389/fpls.2021.775383

**Published:** 2022-01-06

**Authors:** Firdissa E. Bokore, Ron E. Knox, Colin W. Hiebert, Richard D. Cuthbert, Ron M. DePauw, Brad Meyer, Amidou N’Diaye, Curtis J. Pozniak, Brent D. McCallum

**Affiliations:** ^1^Swift Current Research and Development Center, Agriculture and Agri-Food Canada (AAFC), Swift Current, SK, Canada; ^2^Morden Research and Development Centre, Agriculture and Agri-Food Canada, Morden, MB, Canada; ^3^Department of Plant Sciences, University of Saskatchewan, Saskatoon, SK, Canada

**Keywords:** *Triticum aestivum*, leaf rust, QTL mapping, SNP markers, gene combination

## Abstract

The hexaploid spring wheat cultivar, Carberry, was registered in Canada in 2009, and has since been grown over an extensive area on the Canadian Prairies. Carberry has maintained a very high level of leaf rust (*Puccinia triticina* Eriks.) resistance since its release. To understand the genetic basis of Carberry’s leaf rust resistance, Carberry was crossed with the susceptible cultivar, Thatcher, and a doubled haploid (DH) population of 297 lines was generated. The DH population was evaluated for leaf rust in seven field environments at the adult plant stage. Seedling and adult plant resistance (APR) to multiple virulence phenotypes of *P. triticina* was evaluated on the parents and the progeny population in controlled greenhouse studies. The population was genotyped with the wheat 90 K iSelect single nucleotide polymorphism (SNP) array, and quantitative trait loci (QTL) analysis was performed. The analysis using field leaf rust response indicated that Carberry contributed nine QTL located on chromosomes 1B, 2B (2 loci), 2D, 4A, 4B, 5A, 5B, and 7D. The QTL located on 1B, 2B, 5B, and 7D chromosomes were observed in two or more environments, whereas the remainder were detected in single environments. The resistance on 1B, detected in five environments, was attributed to *Lr46* and on 7D, detected in seven environments to *Lr34*. The first 2B QTL corresponded with the adult plant gene, *Lr13*, while the second QTL corresponded with *Lr16*. The seedling analysis showed that Carberry carries *Lr2a*, *Lr16*, and *Lr23*. Five epistatic effects were identified in the population, with synergistic interactions being observed for *Lr34* with *Lr46*, *Lr16*, and *Lr2a*. The durable rust resistance of Carberry is attributed to *Lr34* and *Lr46* in combination with these other resistance genes, because the resistance has remained effective even though the *P. triticina* population has evolved virulent to *Lr2a, Lr13, Lr16*, and *Lr23*.

## Introduction

Wheat (*Triticum aestivum* L.) is the most widely cultivated crop globally, and it is a major source of calories and protein for the world population (Shiferaw et al., 2013; Shewry and Hey, 2015). Significant constraints to increased wheat production in Canada and internationally are the rust diseases, such as leaf rust (*Puccinia triticina* Eriks.), stem rust (*Puccinia graminis* Pers.: Pers. f. sp. *Tritici* Eriks. & E. Henn.), and stripe rust (*Puccinia striiformis* Westend. f. sp. *tritici*) (Mcintosh et al., 1995; Kolmer, 2005; McCallum et al., 2007). Historically, leaf rust had caused major crop losses in North America (Peturson, 1958; Kolmer, 2005; McCallum et al., 2007). Leaf rust was abundant in the Prairie Provinces of Canada in 1953, 1954, and 1955, and it was prevalent and well established in the region somewhat later than stem rust (Peturson, 1958). Considerable rust damage was caused in all 3 years but particularly in 1954, when both leaf and stem rusts were heavy throughout most of Manitoba and Saskatchewan and in a considerable area in the east-central Alberta. Corresponding wheat yield reduction in the Western Canada due to leaf rust and stem rust was estimated at about 45 million bushels in 1953, 150 million bushels in 1954, and 9 million bushels in 1955. Leaf rust was also a production problem in the 1980s when the cultivar AC Barrie was the dominant wheat cultivar in the western Canada (McCallum and DePauw, 2008). Leaf rust can still pose a serious threat to wheat production if breeding for resistance and other management practices are relaxed (Aboukhaddour et al., 2020).

Over time, improved resistance to leaf rust was achieved in western Canada by developing cultivars with additional genes for resistance (McCallum and DePauw, 2008). For example, a hard red spring wheat cultivar, Thatcher was susceptible to leaf rust, although it was the first significant stem rust resistant cultivar grown in Canada extensively over a long period of time extending from 1939 to 1968 (McCallum et al., 2007; McCallum and DePauw, 2008). Thatcher was selected from a double cross Marquis/Iumillo//Marquis/Kanred wheat in 1925 and released in 1935 (Hayes et al., 1936). The resistant durum variety, Iumillo, and the winter wheat variety, Kanred are the ancestors of Thatcher, from which it inherited some of its resistance (Hayes et al., 1936). However, Thatcher is generally very susceptible to leaf rust, except to a few races, and has been used as a universal susceptible genetic background to develop the Thatcher near isogenic wheat lines (McCallum et al., 2016). Using a cross of a Romanian wheat line Fundulea 900 and Thatcher, Zhang et al. (2017) reported a minor effect of leaf rust resistance QTL on chromosome 2DS, *QLr.hebau-2DS* contributed by Thatcher. Zhang et al. (2017) additionally indicated that *Lr22b* may confer residual resistance in field nurseries when challenged with isolates virulent on *Lr22b*, or another gene linked to *Lr22b* confers this resistance from Thatcher. Previously, Dyck (1979) indicated that Thatcher carries *Lr22b* which confers adult plant resistance (APR) to leaf rust to only a few virulence phenotypes.

Carberry is a semi-dwarf doubled haploid (DH), hard red spring wheat variety that is derived from the cross, Alsen by Superb made in 2000 at the Swift Current Research and Development Centre, AAFC, SK, Canada and registered in 2009 (DePauw et al., 2011). It was grown over 2.3 Mha in the years 2011--2019^1^. Carberry was resistant to both leaf rust and stem rust at the time of its release (DePauw et al., 2011) and currently, it still has resistance to both rust diseases and moderate resistance to stripe rust. Superb has the resistance genes, *Lr2a* and *Lr10* (McCallum and Seto-Goh, 2010), and Alsen is reported to have genes, *Lr2a*, *Lr10, Lr13, Lr23*, and *Lr34* (Oelke and Kolmer, 2005).

Resistance has been and will continue to be the major means for controlling cereal rusts (Roelfs, 1988). Two classes of genes, namely all stage (seedling) resistance (ASR) and APR are known. ASR is expressed throughout the life of the plant, whereas APR is expressed only at later stages in the plant’s development (Ellis et al., 2014; da Silva et al., 2018). Most of the designated genes confer ASR against leaf rust and some QTL, reported in different genetic backgrounds, provide APR (McIntosh et al., 2014). *Lr34* and *Sr2* are APR genes that have been deployed in conjunction with other ASR and APR genes providing resistance in wheat cultivars widely grown over many years, therefore, demonstrating durable resistance (Ellis et al., 2014). ASR is governed by major or race-specific genes and is often characterized by its short longevity as compared to some APR genes. For example, the ASR gene, *Lr10* became ineffective and *Lr16* became partially ineffective within a few years of their deployment in Canadian wheat cultivar, Selkirk (da Silva et al., 2018). In Canada, improved leaf resistance has been achieved primarily due to the use of genes, such as *Lr13*, *Lr14a*, *Lr16*, *Lr21*, and *Lr34* (McCallum and DePauw, 2008; McCallum et al., 2016).

Breeding for resistance has evolved with the advent of molecular mapping technologies and the development of markers linked with resistance genes. Identifying and mapping genes, and developing genetic markers for marker-assisted breeding is helpful to develop wheat varieties with an acceptable level of resistance. This study was conducted to understand the genetic basis of leaf rust resistance in Carberry through a cross with the susceptible variety, Thatcher.

## Materials and Methods

### Plant Material

A DH population of 297 lines generated from F_1_ plants of a cross Carberry/Thatcher (CT) was used in this study. Carberry is resistant to leaf rust (DePauw et al., 2011), whereas Thatcher is susceptible (Dyck et al., 1966). The population was evaluated for ASR in the greenhouse and APR in the greenhouse and in the field.

### Disease Evaluation

#### Seedling Leaf Rust Analysis

To determine the number and identity of the all stage (seedling) leaf rust resistance genes in Carberry, the parents and the CT population were inoculated at the seedling stage with multiple virulent phenotypes of *P. triticina*. Urediniospores of single purified isolates of *P. triticina* were used to inoculate this population at the two-leaf growth stage as described by McCallum et al. (2020). The isolates used included 1-1 BBBD, 96-12-3 MBDS, 128-1 MBRJ, 74-2 MGBJ, 11-180-1 TDBG, 06-1-1 TDBG, 77-2 TJBJ, 9-1 SBDG, 161-1 FBDS, 08-5-1 TDBB,19-129-1 TBTN, and 18-10-1 TBBS (McCallum et al., 2020). The race letter codes used in the isolate names define the virulence/avirulence formula of each isolate as described by Long and Kolmer (1989). Plants were rated to determine the infection type 12–14 days post-inoculation (McCallum et al., 2020). Host lines that produced infection types “;” (hypersensitive flecks), “1” (small uredinia with necrosis), and “2” (small to medium sized uredinia with chlorosis) were considered resistant, and those that produced infection types “3” (medium sized uredinia without chlorosis or necrosis) and “4” (large uredinia without chlorosis or necrosis) were considered susceptible.

#### Determination of Leaf Rust Resistance in Superb and Alsen, the Parental Lines of Carberry

From the initial seedling tests, Carberry appeared to have the resistance gene, *Lr16*, which was not reported in either of its parents, Superb and Alsen, and appeared to lack *Lr10* that was reported to be present in both the parental lines (Oelke and Kolmer, 2005; McCallum and Seto-Goh, 2010). Seeds of the parental lines which were used to make the cross that resulted in Carberry were tested for seedling leaf rust resistance and the presence or absence of molecular markers associated with *Lr16*. Sixteen seeds of each parental line (Superb and Alsen) were planted in individual root trainers. The check lines, Carberry, Thatcher, the Thatcher near isogenic lines with *Lr2a*, *Lr10*, *Lr16*, and *Lr23* and the standard set of 16 North American leaf rust differential lines were also planted. These plants were then inoculated with the isolate, 20-140-1 TBRD, which is virulent to *Lr2a* and *Lr23* but avirulent to *Lr10* and *Lr16*. Then a second set of plants, as described above, was inoculated with the isolate, 19-123-2 TBGJ which is virulent to *Lr2a*, *Lr23*, and *Lr10*, but avirulent to *Lr16*.

Leaf tissue was sampled from Superb, Alsen, and check lines, and the DNA was extracted. Carberry, Superb, Alsen, and check lines were genotyped with two Kompetitive Allele-Specific PCR (KASP) markers, *kwm677* and *kwm849* that are diagnostic of *Lr16* (Kassa et al., 2017). KASP assays were performed as described by Kassa et al. (2017).

#### *Lr13* Adult Plant Resistance Evaluation

To determine the presence or absence of *Lr13* in Carberry, it was grown along with Thatcher and the CT population in square pots (15 cm) in the greenhouse, and one pot was inoculated with each of the *Lr13* avirulent isolates 16-284-1 TGBQ and 17-358-1 TBBJ at the flag leaf stage, as described by McCallum et al. (2020). Both isolates were virulent to *Lr2a* and *Lr23*, but had intermediate (16-284-1 TGBQ) or avirulent (17-358-1 TBBJ) reactions to *Lr16*. Plants were rated 14 days later for the infection type produced and classified as resistant or susceptible as described above. Although both *Lr34* and *Lr46* APR genes were present in this population, the infection type for *Lr13* was lower or more resistant than that produced by either of these genes, allowing for the identification of *Lr13* in the presence of *Lr34* and/or *Lr46*.

#### Hybrid Necrosis Test to Determine the Presence of *Lr13* in Carberry

Leaf rust resistance *Lr13* and progressive necrosis *Ne2m* are conditioned by a single pleiotropic gene (Zhang et al., 2016). When *Lr13*/*Ne2m* carriers are crossed with *Ne1* carriers, the F_1_ progeny show the distinctive phenotype of progressive necrosis where leaves, starting with the first leaf, undergo necrosis and die-off as the plant develops. To confirm the presence of *Lr13* in Carberry, Carberry was crossed with Kubanka, tetraploid wheat that is a carrier of *Ne1*. The F_1_ progeny were grown in conditions as described above and were observed for progressive necrosis once the third to the fourth leaves fully emerged. Zhang et al. (2016) mapped *Lr13* and *Ne2* using a DH population from the cross Thatcher/Thatcher-*Lr13*. DH lines were crossed with Kubanka in their study to confirm the cosegregation of *Lr13* and *Ne2*. F_1_ progeny from these crosses with Kubanka were used as positive (*Lr13* present) and negative (*Lr13* absent) controls for the presence of progressive necrosis.

#### Field Trials

The field trials were conducted near Swift Current, SK in 2014, 2015, 2016, and 2018 and at Morden, MB in 2016, 2019, and 2020. Entries were planted in single 1 m rows in groups of five flanked by susceptible spreader rows. At Morden, the experiments consisted of two replications in a randomized complete block design, whereas the trials at Swift Current were planted as single entries with repeated parents and checks. Given the population size of nearly 300 lines, each allele at each locus is replicated roughly 150 times in the population that behaves as a diploid. The spreader rows were inoculated with a mixture of leaf rust races for ease of disease development and infection of entries as previously explained in Bokore et al. (2020). Briefly, the inoculum of *P. triticina* was generated by increasing the urediniospores of all races in the proportions they were found in the western Canada in the year prior to the field trial. Urediniospores of these multi-race mixtures were used to inoculate spreader rows susceptible to leaf rust at both Swift Current and Morden. For each year, all the isolates generated during the virulence survey of Manitoba and Saskatchewan were combined to generate this field inoculum. In each year, the same *P. triticina* race composition was used in Morden and Swift Current trials.

At the Morden location, urediniospores were suspended in light mineral oil (Soltrol, Chevron Phillips Chemical Company) and sprayed on the leaves of the spreader rows at early tillering. Subsequently, leaf rust developed on the spreader rows and urediniospores were windblown to the test lines to provide infection. At Swift Current, spreader rows of susceptible genotypes were needle inoculated with leaf rust urediniospores (Bokore et al., 2020). Irrigation misting was used to provide conditions suitable for the development and spread of leaf rust. Leaf rust severity was scored from 0 to 100% using the modified Cobb Scale (Peterson et al., 1948). Infection response (IR) was recorded as resistant (R), resistant to moderately resistant (RMR), moderately resistant (MR), mesothetic (X), moderately resistant to moderately susceptible (MRMS), moderately susceptible (MS), moderately susceptible to susceptible (MSS), and susceptible (S). To utilize the data for the analysis of main effect QTL, the epistatic effects, and the infection response scores were converted to numerical values as *R* = 1, RMR = 2, MR = 3, *X* = 4, MRMS = 5, MS = 6, MSS = 7, and *S* = 8. Simple means of two replications of each experiment were used for the QTL analysis at Morden, whereas single plot data was used for the QTL analysis at Swift Current.

### Genotyping and Linkage Mapping

The DNAs of the parents and 297 lines were extracted from young leaves using the DNeasy 96 Plant Kit (QIAGEN Science, MD, United States). The lines and parents were genotyped with the wheat 90K iSelect single nucleotide polymorphism (SNP) genotyping array (Illumina Inc., San Diego, CA). The raw data were processed using GenomeStudio v2.0 software (Illumina). Of the 81,587 SNPs contained on the 90K iSelect SNP genotyping array, 8,360 high quality polymorphic SNPs were identified. The SNPs were identified by filtering to include only those with two major cluster frequencies displaying near 1:1 segregation expected for a DH population (each cluster containing >35 and <65% of total lines) with the third cluster containing a maximum 5% of total lines. SNPs were further filtered to include only cluster plots with a high (>0.6) GenTrain Score (the GenomeStudio clustering algorithm measuring SNP calling quality ranging from 0 to 1). Finally, only SNPs with a high (>90%) call frequency were accepted. The resulting SNP calls were then exported to MS Excel and converted to a binary mapping matrix by phasing the SNP calls corresponding to Thatcher as “A” and the SNP calls corresponding to Carberry as “B” for each SNP.

The genetic map was built using a two-step strategy as previously described by Fowler et al. (2016) and Perez-Lara et al. (2016). First, markers were clustered into linkage groups with a stringent cut off *p*-value of 1^–10^ and a maximum distance between markers of 15 cM, using the minimum spanning tree map (MSTMap) software (Wu et al., 2008). Next, the linkage groups were refined using the MapDisto version 1.7.5 software (Lorieux, 2012) using a cut off recombination value of 0.35, a minimum logarithm of odds (LOD) score of 3.0, and a Kosambi mapping function (Kosambi, 1944). The best order of markers was generated using both “AutoCheckInversions” and “AutoRipple” commands. Linkage groups were assigned to their belonging chromosomes based on the existing high density SNP maps of wheat (Cavanagh et al., 2013; Maccaferri et al., 2014, Wang et al., 2014).

### Statistical Analysis

Pearson’s correlation coefficients, among disease data of different environments, were calculated using the CORR procedure of SAS v.9.3 (SAS Institute, Cary, NC, United States). Broad-sense heritability and narrow sense heritability of the disease resistance were calculated by QTLNetwork 2.0 (Yang et al., 2005, 2008). As the population used in the present study was DH, dominance gene effects were absent. Broad-sense heritability was calculated as the variance of genetic main effects divided by phenotypic variance [V(G)/V(P)], whereas narrow sense heritability was calculated as the variance of additive genetic effect divided by phenotypic variance [V(A)/V(P)]. The epistasis heritability was calculated as the variance of additive × additive divided by phenotypic variance [V(AA)/V(P)]. To compare single gene effects with combined gene effects, we performed the analysis of variance and Duncan’s Multiple Range Test (DMRT) with SAS software (SAS Institute, NC v.9.3). The comparison among gene combination effects focused on a pool of four genes *Lr2a*, *Lr16*, *Lr34* and *Lr46*, present in Carberry. To perform the combined gene effect analysis, the DH lines were sorted into different classes based on markers that were associated with each gene. *Lr13*, while not effective on its own in the field, may have some degree of interaction and a background effect through its interaction with the many other resistance genes present in this population. As the number of interactions gets to be large, when these many effective genes are involved, *Lr13* was not included in the combined analysis.

### Detection of Main and Epistatic Quantitative Trait Loci Effects Using Field Data

The analysis of the main effect of QTL was carried out for each environment on DS and IR data, and on seedling infection response data by MapQTL 6 software, Kyazma (Van Ooijen, 2009). The QTL analysis performed based on the seedling infection response was used to compare with the QTL identified using the field data (results not presented). Simple interval mapping followed by multiple QTL mapping (MQM) approaches were conducted to detect the main effect of QTL. Cofactor markers were selected using automatic cofactor selection based on the backward elimination of markers and/or adjusted by selecting a set of markers manually. To determine the significant threshold of LOD values, a permutation test of 1,000 iterations was performed. The significance of each QTL was declared at 5% probability.

To determine the epistatic interactions between the main effect QTL, an epistasis analysis was performed by QTLNetwork 2.0, which was used to detect single-locus and epistatic QTL simultaneously (Yang et al., 2005, 2008). Mixed-model-based composite interval mapping (MCIM) within QTLNetwork 2.0 was selected for a one-dimensional (1D) genome scan to search for single-locus QTL. To determine epistatic effects, a two-dimensional (2D) genome scan procedure was used. The main effect QTL and epistatic interaction were declared significant at 5% probability.

## Results

### Greenhouse Leaf Rust Reaction Analysis

All isolates tested were virulent on Thatcher and avirulent on Carberry (Table 1). The segregation ratio was consistent with a single resistance gene when the progeny lines were inoculated with 11-180-1 TDBG, 06-1-1 TDBG, 95-77-2 TJBJ, and 18-10-1 TBBS (Table 2). This resistance gene gave a ‘1+’ infection type, characteristic of the reaction of these isolates to the Thatcher-*Lr16* line (Table 1). Each of the progeny lines was scored as having the resistant or susceptible allele for this gene and the results for each of these virulent phenotypes were mapped as QTL for seedling leaf rust resistance in the *Lr16* region of chromosome 2BS. This gene was also effective against all the other virulent phenotypes consistent with their avirulent response to *Lr16* [though both 95-74-2 MGBJ and 95-77-2 TJBJ had intermediate pustule types on lines with *Lr16* (Table 1)].

**TABLE 1:** *Puccinia triticina* isolates used for seedling test and the reaction of known wheat lines against each isolate.

	*Puccinia triticina* Isolate									
Line	1-1 BBBD	96-12-3 MBDS	94-128-1 MBRJ	95-74-2 MGBJ	11-180-1 TDBG	06-1-1 TDBG	95-77-2 TJBJ	9-1 SBDG	161-1 FBDS	18-10-1 TBBS
Carberry	0^b^	0	0;	0	1+	1+	13^c^	;	0	1+
Thatcher	3	3	3	3	3+	3+	3	3	3	3
Tc-*Lr2a*^a^	0;	0;	0	0	3+	3	3	3+	;1=	3
Tc-*Lr16*	;1=	1 1−	1−	1 3	1+	1+	1 3−	;1−	1+	1+
Tc-*Lr23*	3 3+	3+	3+	3+	3+	3+	3+	;1− 2−	2	3
Tc-*Lr10*	;1=	3	3+	3+	3	3	3	3	3	3

*^a^The Thatcher near isogenic wheat lines used included RL (Tc-Lr2a), RL (Tc-Lr16), RL (Tc-Lr23), RL (Tc-Lr10).*

*^b^Host lines that produced infection types “;” (hypersensitive flecks), “1” (small uredinia with necrosis), and “2” (small- to medium-sized uredinia with chlorosis) were considered resistant, and those that produced infection types “3” (medium sized uredinia without chlorosis or necrosis) were considered susceptible. Pustules larger than normal for the infection type were indicated with “+” and those smaller were indicated with a “−” or a “=” for very small pustules.*

*^c^In some cases, a range of pustule types were observed on the same leaves, and these are listed with a space between each different pustule type.*

**TABLE 2:** Reaction of the Carberry/Thatcher population against *P. triticina* isolates at the seedling stage.

Isolate	Gene(s) detected	Progeny Lines		Expected ratio	*X* ^2^	*P*-value
		Resistant	Susceptible			
11-180-1 TDBG^a^	*Lr16*	157	141	1:1	0.86	0.35
96-12-3 MBDS^b^	*Lr2a*, *Lr16*	217	80	3:1	0.59	0.44
9-1 SBDG	*Lr16*, *Lr23*	215	83	3:1	1.29	0.26
161-1 FBDS	*Lr2a*, *Lr16*, *Lr23*	278	27	7:1	3.70	0.03

*^a^Progeny lines reacted the same to 11-180-1 TDBG, 06-1-1 TDBG, 95-77-2 TJBJ, and 18-10-1 TBBS.*

*^b^Progeny lines reacted the same to 96-12-3 MBDS, 94-128-1 MBRJ, and 95-74-2 MGBJ.*

When 96-12-3 MBDS, 94-128-1 MBRJ, and 95-74-2 MGBJ were inoculated onto the progeny lines, the segregation ratios were consistent with two effective resistance genes, *Lr16* and a second resistance gene thought to be *Lr2a* since it was effective against isolates avirulent to *Lr2a* and ineffective to isolates virulent to *Lr2a* (Table 2). This second gene had a very resistant infection type consistent with *Lr2a* (Table 1) which made the determination of the presence or absence of this gene possible in the presence of *Lr16*. All the progeny lines were scored for the presence or absence of this resistance gene and it was mapped as a QTL for seedling leaf rust resistance to the *Lr2* region of chromosome 2DS.

The segregation ratio of the progeny lines to 9-1 SBDG also indicated the presence of two resistance genes, one of which was *Lr16*; however, this isolate is virulent to *Lr2a*, so a third seedling resistance gene was also present. This third resistance gene was ineffective against all the isolates tested except 9-1 SBDG and 161-FBDS, which were the only isolates avirulent to *Lr23*. When progeny lines were scored for the presence or absence of this resistance gene, the results also mapped to chromosome 2BS and is thought to be *Lr23*, which was reported to be present in Alsen, one of the parents of Carberry (Oelke and Kolmer, 2005). The population appeared to segregate three resistance genes to 161-1 FBDS, *Lr16*, *Lr2a*, and *Lr23*, fitting both a three and four gene ratio. This third gene (*Lr23*) also appeared to have some effect on 1-1 BBBD but this reaction could not be determined as consistently as it could when these lines were inoculated with 9-1 SBDG or 161-FBDS.

The seedling resistance gene, *Lr10* was reported to be present in both the parents of Carberry (Oelke and Kolmer, 2005; McCallum and Seto-Goh, 2010). When isolates, 08-5-1 TDBB and 19-129-1 TBTN, both of which were avirulent on *Lr10*, were inoculated onto the progeny, the reactions were the same as to 11-180-1 TDBG, 06-1-1 TDBG, 95-77-2 TJBJ, and 18-10-1 TBBS, indicating the presence of *Lr16*, but with no additional resistance gene. From this, it appears that *Lr10* is not present in Carberry.

#### Determination of Leaf Rust Resistance in Superb and Alsen, the Parental Lines for Carberry

When plants of the parental lines of Carberry (Superb and Alsen) were inoculated with the isolate 20-140-1 TBRD, which was virulent to *Lr2a* and *Lr23* but avirulent to *Lr10* and *Lr16*, all 16 Superb plants were resistant with a consistent infection type of “11+ 2−“ characteristic of *Lr10*. The Alsen plants, however, varied in their response to this isolate with 14 resistant plants and two susceptible plants. This demonstrates that at least some plants of Alsen did not have *Lr10*. When a second set of plants was inoculated with the isolate, 19-123-2 TBGJ, which was virulent to *Lr2a*, *Lr23*, and *Lr10*, but avirulent to *Lr16*, all the Superb plants were uniformly susceptible, but the Alsen plants again varied for their infection types with nine resistant and seven susceptible plants.

When these same plants were marker tested, marker alleles corresponded with resistance to races, 20-140-1 TBRD and 19-123-2 TBGJ. The data showed that Carberry carries *Lr16*, Superb lacks *Lr16*, and Alsen is heterogeneous for *Lr16*. The presence of *Lr16* in the Alsen plants corresponded with a resistant infection type when inoculated with the *P. triticina* isolate, 19-123-2 TBGJ. The phenotypic and genotypic data confirm that Carberry carries *Lr16* inherited from Alsen (Supplementary Table 1).

#### *Lr13* Adult Plant Resistance Evaluation

When adult plants of Carberry, Thatcher, and each line in the CT population were inoculated at the adult plant stage with each of the isolates, 16-284-1 TGBQ and 17-358-1 TBBJ, Carberry was resistant and Thatcher was susceptible. The progeny lines segregated for resistance with similar reactions to both isolates. All progeny lines with *Lr16* were resistant to both the isolates. Even though 16-284-1 TGBQ is classified as virulent on *Lr16*, the reaction is intermediate, and host plants were more resistant at the adult plant stage than at the seedling stage. For the progeny lines without *Lr16*, 50 were resistant and 87 were susceptible, which indicated the presence of another resistance gene, likely *Lr13*, but the results did not fit a single gene segregation ratio. Even though both *Lr34* and *Lr46* were present in this population, they did not affect the detection of *Lr13* since plants with *Lr13* had uniform infection types of “1” or ‘1–2,” whereas *Lr34* and *Lr46* produce infection types with mixtures of pustule types, including “3” or susceptible pustules.

#### Hybrid Necrosis Test to Determine the Presence of *Lr13* in Carberry

**FIGURE 1:**
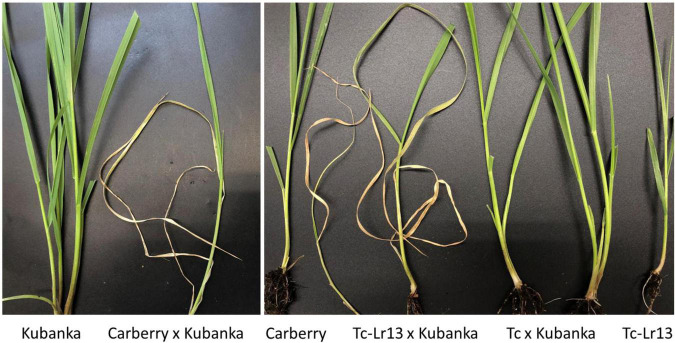
Seedlings of Kubanka, Carberry, Thatcher *Lr13* (Tc-*Lr13*) and F_1_ of Carberry × Kubanka, Tc-*Lr13* × Kubanka and Thatcher (Tc) × Kubanka. Hybrid necrosis in the Carberry × Kubanka F1 generation lines confirms the presence of *Lr13* in Carberry.

Seedlings of the parental lines of Carberry, Thatcher, Thatcher-*Lr13*, and Kubanka grew normally with healthy green leaves all the way to early tillering. Similarly, hybrids between Thatcher/Thatcher-*Lr13* DH lines that lacked *Lr13* and Kubanka showed the same healthy growth pattern. Hybrids between Carberry and Kubanka and between Thatcher/Thatcher-*Lr13* DH lines carrying *Lr13* and Kubanka showed strong progressive necrosis (Figure 1). Necrosis of the first leaf became evident once the third leaf had fully emerged.

### Disease Evaluation in the Field

The resistant parent Carberry conferred high resistance to leaf rust with low disease severity (DS) and IR ranging from R to MR across environments, whereas the susceptible parent Thatcher had high DS and IR ranging from MSS to S (Table 3). The disease severity of the CT population ranged from 0.0 to 90% across environments at Morden and from 0.5 to 80% across environments at Swift Current. Although Swift Current across years had lower disease scores than Morden, the disease pressure observed in each environment was sufficient for discriminating among the lines and mapping loci associated with quantitative resistance. A wide range of heritability values of disease response was observed in the population (Table 3). Broad sense heritability of DS ranged from 0.25 to 0.63, and of IR from 0.19 to 0.54 across environments. Furthermore, narrow sense heritability ranged from 0.22 to 0.61 for the DS and from 0.18 to 0.51 for the IR.

**TABLE 3:** Mean and range of the leaf rust severity and infection response scores for the Carberry/Thatcher doubled haploid (DH) population and parents and heritabilities from field nurseries near Morden (MD), MB for 3 years, and Swift Current (SC), SK for 5 years.

Location-Year	Population			Carberry	Thatcher	Components of variance for disease severity^a^			
	Min	Max	Mean			V(G)/V(P)	V(A)/V(P)	V(AA)/V(P)	V(e)/V(P)
**Disease severity (%):**									
MD2016	0.0	90.0	38.6	3.9	75.7	0.61	0.59	0.02	0.39
MD2019	0.0	87.5	31.5	2.1	65.9	0.53	0.51	0.02	0.47
MD2020	0.0	90	28.2	0.0	79.3	0.63	0.61	0.02	0.37
SC2014	0.5	60	15.4	3.1	33.5	0.39	0.36	0.03	0.61
SC2015	0.5	60	14.5	4.1	29.9	0.29	0.29	−	0.71
SC2016	0.5	80	16.2	1.3	45.1	0.43	0.40	0.02	0.57
SC2018	0.5	80	4.5	0.2	18.0	0.25	0.22	0.03	0.75
**Infection response^b^**									
MD2016	R	S		R	S	0.54	0.51	0.04	0.46
MD2019	R	S		R	S	0.44	0.43	0.01	0.56
MD2020	R	S		R	S	0.36	0.36	−	0.64
SC2014	RMR	S		MR	MSS	0.35	0.30	0.05	0.65
SC2015	R	S		MR	MSS	0.40	0.35	0.05	0.60
SC2016	R	S		MR	MSS	0.19	0.18	0.01	0.81
SC2018	R	S		RMR	MSS	0.25	0.25	−	0.75

*^a^V(G), genotype variance; V(A), additive variance; AA, additive × additive variance; V(P), phenotypic variance; V(G)/V(P), variance of genetic main effects divided by phenotypic variance (broad sense heritability); V(A)/V(P), narrow sense heritability; V(AA)/V(P), additive × additive epistasis heritability; V(e)/V(P), variance of residual effects divided by phenotypic variance.*

*^b^R, resistant; RMR, resistant to moderately resistant; MR, moderately resistant; MSS, moderately susceptible to susceptible; S, susceptible.*

**FIGURE 2:**
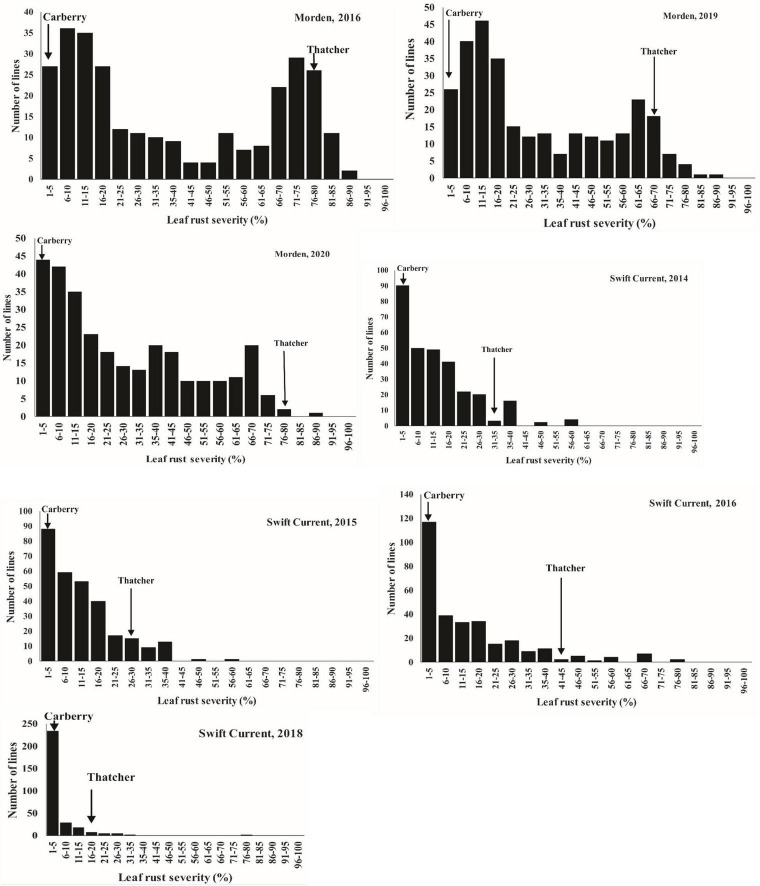
Distributions of leaf rust severity for Carberry/Thatcher lines evaluated over multiple years in Morden, MB and Swift Current, SK locations. Arrows indicate the severity of the parents.

Except at Morden in 2016 and 2019, which displayed bimodal distributions, the DS of the population was continuous with a preponderance of lines showing low-disease scores (Figure 2). When the most resistant (0 to <20%) and susceptible (>60 to <90%) portions of the distribution tails for leaf rust severity were considered, markers showed a disproportionate representation for *Lr34* for the Morden 2019 and 2016 environments. In the Morden 2019 environment, out of 120 lines in the resistant mode, 99 of them carried the *Lr34* resistance allele compared with 21 of the lines which did not carry the resistance allele. In the susceptible mode, only 6 out of 39 lines possessed the *Lr34* resistance allele. The same trend was observed in the Morden 2016 environment in which 84 out of 103 lines in the resistant mode had the *Lr34* resistance allele, while 19 lines did not. In the susceptible mode, from a total of 39 lines, only 6 lines had the *Lr34* resistance allele. Correlation coefficients (Table 4) among environments for disease severity were highly significant and ranged from moderate to high (*r* = 0.54 to 0.90, *P* < *0.0001*).

**TABLE 4:** Pearson correlation coefficients (r) of the relation between leaf rust severities of the DH lines of the Carberry/Thatcher population evaluated near Morden (MD) and Swift Current (SC) from 2014 to 2020.

	Leaf rust severity (%)					
	SC 2015	SC 2016	SC 2018	MD 2016	MD 2019	MD 2020
SC 2014	0.62^a^	0.64	0.64	0.69	0.68	0.72
SC 2015	−	0.56	0.68	0.64	0.60	0.67
SC 2016		−	0.57	0.70	0.71	0.72
SC 2018			−	0.55	0.54	0.63
MD 2016				−	0.89	0.88
MD2019					−	0.90

*^a^The correlation coefficients were significant at P < 0.0001.*

### Linkage Map

The genetic map of the CT population consisted of 8,360 polymorphic SNP markers (Supplementary Table 2). The map covered 3645.8 cM of the wheat genome, corresponding to an average density of 0.44 cM per marker. All of the 21 wheat chromosomes, except 4D, were represented in 28 linkage groups.

### Leaf Rust Resistance Quantitative Trait Loci Identified

**FIGURE 3:**
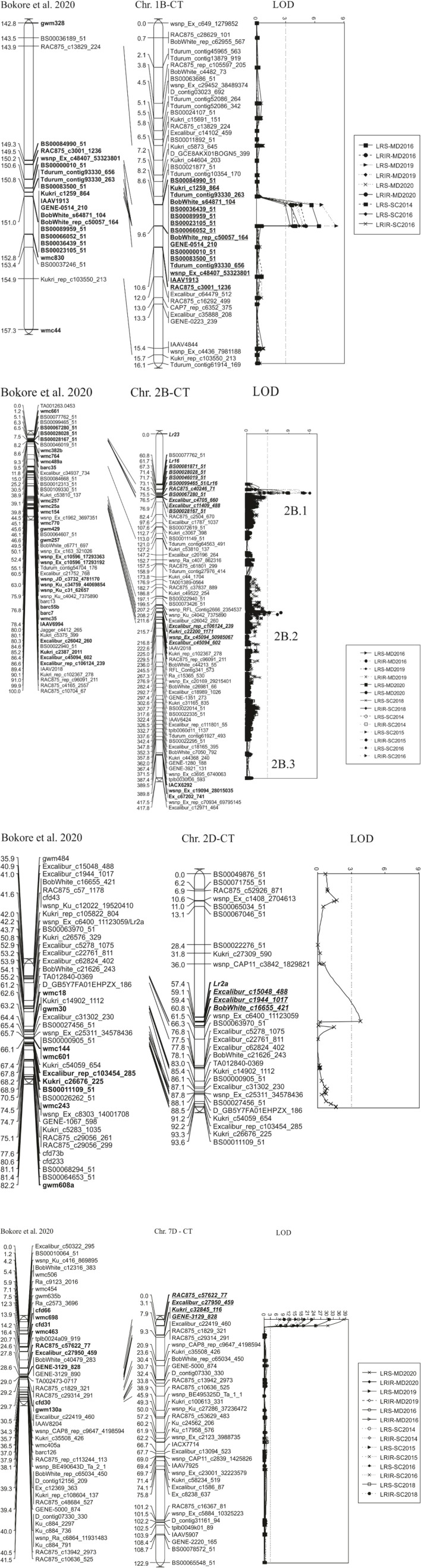
Quantitative trait loci (QTL) for leaf rust resistance identified in Carberry/Thatcher (CT) population evaluated in seven field environments at the adult plant stage near Morden (MD), MB and Swift Current (SC), SK, Canada. QTL detected in the population on chromosome 4A, 4B, and 5B were not presented in graphs. Note that *2B.1* stands for *QLr.spa-2B.1, 2B.2* for *QLr.spa-2B.2*, and *2B.3* for *QLr.spa-2B.3. QLr.spa-2B.*3 was not detected by MapQTL and QTLNetwork as the main effect QTL, but it was revealed by epistasis analysis interacting with *QLr.spa-2D*. Disease traits, leaf rust severity (LRS) and leaf rust infection response (LRIR), and test locations Morden (MD) and Swift Current (SC) and the year of field evaluation. The Carberry/Thatcher (CT) population map was aligned with a hexaploid high density consensus map published by Bokore et al. (2020).

Overall, nine QTL associated with field data were detected on chromosomes, 1B (designated *QLr*.*spa-1B*), 2B [2 loci (*QLr.spa-2B.1; QLr.spa-2B.2*)], 2D (*QLr.spa-2D*), 4A (*QLr.spa-4A*), 4B (*QLr.spa-4B*), 5A (*QLr.spa-5A*), 5B (*QLr.spa-5B*), and 7D (*QLr.spa-7D*) (Table 5 and Figure 3). Besides their effectiveness at the adult plant stage, *QLr.spa-2B.1* and *QLr.spa-2D* were significant at the seedling stage. Four of the QTL located on 1B, 2B (2 loci), and 7D were detected in multiple environments, whereas the remaining were detected in one or a maximum of two out of seven test environments. Carberry contributed to the desirable alleles at all the detected loci, and no QTL was obtained from Thatcher. Based on the position of QTL- associated markers in a hexaploid wheat high density 90K SNP map by Wang et al. (2014), the identified QTLs were assigned with the chromosome arms, 1BL, 2BS (2 loci), 2DS, 4AS, 4BS, 5AL, 5BS, and 7DS.

**TABLE 5:** Quantitative trait loci (QTL), their chromosome arm location, peak associated marker and LOD, position on the chromosome in centiMorgans (cM), phenotypic value associated with the parental type allele, percent of phenotypic variation explained (PVE%), and additive effect associated with response to leaf rust disease severity and infection response detected in the Carberry/Thatcher population evaluated in field nurseries near Morden, MB, and Swift Current, SK.

Environment	Trait^a^	Chromosome^b^	QTL	Marker	Position, cM	LOD	Thatcher	Carberry	PVE,%	Additive effect^c^
			
							allele value	allele value		
Swift Current 2014	DS	1BL	*QLr.spa-1B*	*BS00000010_51*	9.6	4.0	19.0	13.1	6.0	2.9
Morden 2016	DS	1BL	*QLr.spa-1B*	*BS00000010_51*	9.6	6.7	49.8	31.3	10.1	9.2
Morden 2016	IR	1BL	*QLr.spa-1B*	*BS00000010_51*	9.6	5.4	6.1	4.9	8.0	0.6
Swift Current 2016	DS	1BL	*QLr.spa-1B*	*BS00000010_51*	9.6	4.3	21.1	12.6	6.4	4.3
Swift Current 2016	IR	1BL	*QLr.spa-1B*	*RAC875_c3001_1236*	10.6	3.8	4.9	4.2	5.7	0.4
Morden 2019	DS	1BL	*QLr.spa-1B*	*BS00000010_51*	9.6	8.3	40.8	24.4	12.0	8.2
Morden 2019	IR	1BL	*QLr.spa-1B*	*BS00000010_51*	9.6	5.0	5.5	4.7	7.5	0.4
Morden 2020	DS	1BL	*QLr.spa-1B*	*RAC875_c3001_1236*	10.6	5.0	35.9	23.5	7.5	6.2
Morden 2020	IR	1BL	*QLr.spa-1B*	*RAC875_c3001_1236*	10.6	3.9	5.4	4.6	5.9	0.4
Swift Current 2014	IR	2BS	*QLr.spa-2B.1*	*BS00028167_51*	16.1	3.4	5.8	5.1	5.0	0.3
Swift Current 2015	DS	2BS	*QLr.spa-2B.1*	*BS00028167_51*	16.1	3.6	17.3	12.4	5.2	2.5
Swift Current 2015	IR	2BS	*QLr.spa-2B.1*	*BS00028167_51*	16.1	9.2	6.0	4.8	12.5	0.6
Morden 2016	DS	2BS	*QLr.spa-2B.1*	*BS00028028_51*	10.6	2.6	45.8	35.1	3.5	5.4
Swift Current 2016	DS	2BS	*QLr.spa-2B.1*	*BS00046019_51*	11.0	5.2	21.1	11.8	7.7	4.6
Swift Current 2016	IR	2BS	*QLr.spa-2B.1*	*BS00046019_51*	11.0	8.0	5.0	4.0	11.6	0.5
Swift Current 2018	DS	2BS	*QLr.spa-2B.1*	*BS00046019_51*	11.0	3.7	6.7	3.0	5.5	1.9
Swift Current 2018	R	2BS	*QLr.spa-2B.1*	*BS00046019_51*	11.0	3.3	4.6	4.0	5.0	0.3
Morden 2019	IR	2BS	*QLr.spa-2B.1*	*BS00028028_51*	10.6	3.6	5.4	4.7	5.5	0.3
Morden 2019	DS	2BS	*QLr.spa-2B.1*	*BS00046019_51*	11.0	3.9	37.3	25.9	5.8	5.7
Morden 2020	IR	2BS	*QLr.spa-2B.1*	*BS00028028_51*	10.6	2.5	5.3	4.8	3.5	0.3
Morden 2020	DS	2BS	*QLr.spa-2B.1*	*BS00046019_51*	11.0	5.9	36.1	23.3	8.0	6.4
Swift Current 2014	DS	2BS	*QLr.spa-2B.2*	*Excalibur_c45094_602*	156.0	3.6	18.4	12.9	5.3	2.7
Swift Current 2014	IR	2BS	*QLr.spa-2B.2*	*Excalibur_c45094_602*	156.0	1.8	5.7	5.2	2.6	0.2
Swift Current 2015	DS	2BS	*QLr.spa-2B.2*	*Excalibur_rep_c106124_239*	150.9	2.7	25.7	3.5	3.8	11.1
Swift Current 2015	IR	2BS	*QLr.spa-2B.2*	*Excalibur_c45094_602*	156.0	5.0	5.8	4.9	6.7	0.4
Morden 2016	DS	2BS	*QLr.spa-2B.2*	*Excalibur_c45094_602*	156.0	3.6	46.6	34	4.8	6.3
Morden 2016	IR	2BS	*QLr.spa-2B.2*	*Excalibur_c45094_602*	156.0	3.8	6.0	5.0	5.1	0.5
Swift Current 2015	DS	2DS	*QLr.spa-2D*	*Excalibur_c15048_488*	59.09	3.8	17.1	12	5.8	2.6
Morden 2020	IR	4AS	*QSr.spa-4A*	*RAC875_rep_c70416_332*	0.0	3.4	5.3	4.7	5.1	0.3
Morden 2019	DS	4BS	*QLr.spa-4B*	*BS00095286_51*	107.2	2.7	37.1	26.9	4.2	5.1
Morden 2019	IR	4BS	*QLr.spa-4B*	*BS00095286_51*	107.2	3.5	5.4	4.7	5.2	0.3
Morden 2019	DS	5AL	*QLr.spa-5A*	*Kukri_rep_c104877_2166*	90.7	2.1	35.9	27.4	3.1	4.2
Morden 2019	IR	5AL	*QLr.spa-5A*	*Kukri_rep_c104877_2166*	90.7	3.1	5.5	4.8	4.7	0.4
Swift Current 2018	IR	5BS	*QLr.spa-5B*	*BS00022525_51*	9.7	4.8	4.7	4.0	7.2	0.4
Morden 2019	IR	5BS	*QLr.spa-5B*	*BS00064042_51*	14.9	3.4	5.6	4.8	5.1	0.4
Morden 2016	DS	7DS	*QLr.spa-7D*	*RAC875_c57622_77*	0.0	34.1	56.2	18.9	41.7	18.7
Morden 2016	IR	7DS	*QLr.spa-7D*	*RAC875_c57622_77*	0.0	34.1	6.6	4.0	41.2	1.3
Morden 2019	DS	7DS	*QLr.spa-7D*	*RAC875_c57622_77*	0.0	31.2	44.8	15.6	38.3	14.6
Morden 2019	IR	7DS	*QLr.spa-7D*	*RAC875_c57622_77*	0.0	22.8	5.8	4.2	29.8	0.8
Morden 2020	DS	7DS	*QLr.spa-7D*	*RAC875_c57622_77*	0.0	38.0	43.0	13.0	44.0	15.0
Morden 2020	IR	7DS	*QLr.spa-7D*	*RAC875_c57622_77*	0.0	19.2	5.6	4.2	25.8	0.7
Swift Current 2014	DS	7DS	*QLr.spa-7D*	*RAC875_c57622_77*	0.0	16.5	20.8	9.4	22.6	5.7
Swift Current 2014	IR	7DS	*QLr.spa-7D*	*RAC875_c57622_77*	0.0	18.4	6.2	4.7	24.9	0.8
Swift Current 2015	DS	7DS	*QLr.spa-7D*	*RAC875_c57622_77*	0.0	15.5	19.3	9.2	21.4	5.0
Swift Current 2015	IR	7DS	*QLr.spa-7D*	*RAC875_c57622_77*	0.0	12.5	6.0	4.6	17.6	0.7
Swift Current 2016	DS	7DS	*QLr.spa-7D*	*RAC875_c57622_77*	0.0	19.3	24.1	7.1	25.8	8.5
Swift Current 2016	IR	7DS	*QLr.spa-7D*	*RAC875_c57622_77*	0.0	7.4	4.9	3.9	10.8	0.5
Swift Current 2018	DS	7DS	*QLr.spa-7D*	*RAC875_c57622_77*	0.0	9.6	7.5	1.6	13.8	3.0
Swift Current 2018	IR	7DS	*QLr.spa-7D*	*RAC875_c57622_77*	0.0	12.0	4.8	3.7	17.0	0.6

*The QTL analysis was performed in MapQTL 6 software.*

*^a^DS, disease severity (%); IR, infection response (0–9 scale).*

*^b^Chromosome arm for each QTL was determined based on peak marker location assigned by Wang et al. (2014).*

*^c^Positive additive values indicate that the resistance allele was derived from Carberry.*

The *QLr*.*spa-1B* accounted for the percent of phenotypic variation explained (PVE) of up to 12.0% for DS and 8% PVE for IR and was associated with peak markers *BS00000010_51* and *RAC875_c3001_1236*, and with a maximum LOD of 8.3 (Table 5). *QLr*.*spa-1B* was consistently detected in five of the seven environments. The *QLr.spa-2B.*1 QTL was detected in all test environments compared to *QLr.spa-2B.2* which was detected in three out of seven environments. Based on the position of QTL-associated markers in the CT population linkage map, *QLr.spa-2B.1* and *QLr.spa-2B.2* were about 145.0 cM distance from each other. *QLr.spa-2B.*1 accounted for a PVE of 3.5 to 12.5% for DS and IR, whereas *QLr.spa-2B.2* accounted for 2.6 to 6.7% PVE for both traits. A QTL analysis performed on the seedling data of avirulent races against *Lr16* (11-180-1 TDBG, 06-1-1 TDBG, 95-77-2 TJBJ, and 18-10-1 TBBS) produced a significant QTL at the same location as *QLr.spa-2B.1* identified with field data.

*QLr.spa-7D* was a major QTL detected at all environments and was associated with the peak marker, *RAC875_c57622_77* with LOD values ranging from 7.4 to 28.0. The QTL accounted for PVE values of up to 44% for DS and 30% for IR. Minor QTL, such as *QLr.spa-2D, QLr.spa-4B*, and *QLr.spa-5A* were confined to single environments. *QLr.spa-4B* and *QLr.spa-5A* were significant for both DS and IR, whereas *QLr.spa-2D* was associated only with DS. A QTL from the analysis of phenotypic data of *Lr2a* avirulent races, 96-12-3 MBDS, 94-128-1 MBRJ, and 95-74-2 MGBJ coincided with *QLr.spa-2D*. The other minor QTL, *QLr.spa-5B* was detected at two environments associated with IR, but not with severity.

### Epistatic Effect Quantitative Trait Loci

A total of five additive by additive epistatic interactions were detected by QTLNetwork analysis (Table 6). The interactions involved QTL that was also identified by MapQTL and an additional QTL on chromosome 2B, designated as *QLr*.*spa-2B.3*, which was not identified by MapQTL. Of the five interactions detected, positive epistatic effects with an enhanced level of disease resistance were observed between *QLr*.*spa-1B* (*Lr46*) and *QLr*.*spa-7D* (*Lr34*), *QLr*.*spa-2B.1* (*Lr16*) and *QLr*.*spa-7D* (*Lr34*), and *QLr*.*spa-2D* (*Lr2a*) and *QLr*.*spa-7D* (*Lr34*). The *QLr*.*spa-7D* (*Lr34*) QTL showed the greatest epistatic effects with *QLr*.*spa-1B* (*Lr46*) and *QLr*.*spa-2B.1* (*Lr16*) and less so with *QLr*.*spa-*2D (*Lr2a*). Negative epistatic effects were detected between *QLr*.*spa-1B* (*Lr46*) and *QLr*.*spa-2B.1* (*Lr16*), and *QLr*.*spa-2B.3* and *QLr*.*spa-2D* (*Lr2a*). The negative association between the alleles at *QLr*.*spa-1B* (*Lr46*) and *QLr*.*spa-2B.1* (*Lr16*), revealed that the *Lr16* interaction with *Lr46* did not act as synergistically as expected. Furthermore, the heritability values for the additive × additive epistasis were very low from 0.01 to 0.05 (Table 6). The most common interaction was between *QLr*.*spa-1B* and *QLr*.*spa-7D*.

**TABLE 6:** Epistatic interactions between Carberry leaf rust resistance genes detected by QTLNetwork in the Carberry/Thatcher population evaluated at Morden, MB for 3 years and Swift Current, SK for 4 years.

Trait	QTL1	Marker interval 1	Interval 1, cM	QTL2	Marker interval 2	Interval 2, cM	AA^a^	*P*-Value	H^2^ (AA)^b^
**Morden 2016**									
DS	*QLr.spa-1B*	*BS00000010_51-RAC875_C3001_1236*	8.5–10.6	*QLr.spa-2B.1*	*BS00081871_51- BS00028167_51*	6.5–16.1	−2.56	0.018	0.007
DS	*QLr.spa-1B*	*BS00000010_51-RAC875_C3001_1236*	8.5–10.6	*QLr.spa-7D*	*RAC875_C57622_77-Excalibur_C27950_459*	0.0–3.59	3.94	0.000	0.017
IR	*QLr.spa-1B*	*BS00084990_51-BS00000010_51*	7.2–10.6	*QLr.spa-7D*	*RAC875_C57622_77-Excalibur_C27950_459*	0.0–3.59	0.39	0.000	0.035
**Morden 2019**									
DS	*QLr.spa-1B*	*EXCALIBUR_C64479_512-CAP7_REP_C6352_375*	10.6–13.0	*QLr.spa-7D*	*RAC875_C57622_77-Excalibur_C27950_459*	0.0–3.59	3.51	0.000	0.022
IR	*QLr.spa-1B*	*BS00000010_51-RAC875_C3001_1236*	8.5–13.0	*QLr.spa-7D*	*RAC875_C57622_77-Excalibur_C27950_459*	0.0–3.59	0.13	0.040	0.008
**Morden 2020**									
DS	*QLr.spa-1B*	*BS00021877_51-BS00084990_51*	7.2–13.0	*QLr.spa-7D*	*RAC875_C57622_77-Excalibur_C27950_459*	0.0–3.59	2.15	0.007	0.008
DS	*QLr.spa-2B.1*	*BS00081871_51- BS00028167_51*	6.5–16.1	*QLr.spa-7D*	*RAC875_C57622_77-Excalibur_C27950_459*	0.0–3.59	2.55	0.001	0.012
***Swift Current 2014***									
DS	*QLr.spa-1B*	*EXCALIBUR_C64479_512-CAP7_REP_C6352_375*	7.5–15.7	*QLr.spa-7D*	*RAC875_C57622_77-Excalibur_C27950_459*	0.0–3.59	2.11	0.000	0.03
IR	*QLr.spa-1B*	*BS00063537_51-RAC875_REP_C105597_205*	0.0–5.5	*QLr.spa-7D*	*RAC875_C57622_77-Excalibur_C27950_459*	0.0–3.59	0.35	0.000	0.054
**Swift Current 2015**									
IR	*QLr.spa-2B.3*	*IACX6292- EX_C67202_741*	328.7–329.1	2D	*TA012840-0369-KUKRI_C14902_1112*	79.1–89.5	−0.37	0.000	0.048
***Swift Current 2016***									
DS	*QLr.spa-1B*	*BS00000010_51-RAC875_C3001_1236*	7.2–13.0	*QLr.spa-7D*	*RAC875_C57622_77-Excalibur_C27950_459*	0.0–3.59	2.05	0.005	0.014
DS	*QLr.spa-2B.1*	*BS00081871_51- BS00028167_51*	6.5–16.1	*QLr.spa-7D*	*RAC875_C57622_77-Excalibur_C27950_459*	0.0–3.59	1.57	0.034	0.008
IR	*QLr.spa-1B*	*TDURUM_CONTIG43162_244-EXCALIBUR_C14102_459*	5.5–6.5	*QLr.spa-7D*	*RAC875_C57622_77-Excalibur_C27950_459*	0.0–3.59	0.16	0.044	0.011
***Swift Current 2018***									
DS	*QLr.spa-2B.1*	*BS00081871_51- BS00028167_51*	6.5–16.1	*QLr.spa-7D*	*RAC875_C57622_77-Excalibur_C27950_459*	0.0–3.59	1.00	0.014	0.017
DS	*QLr.spa-2D*	*EXCALIBUR_C1944_1017-BOBWHITE_C16655_421*	51.0–81.1	*QLr.spa-7D*	*RAC875_C57622_77-Excalibur_C27950_459*	0.0–3.59	0.96	0.017	0.016

*^a^AA, Epistasis effect.*

*^b^H^2^(AA), epistatic QTL heritability.*

**FIGURE 4:**
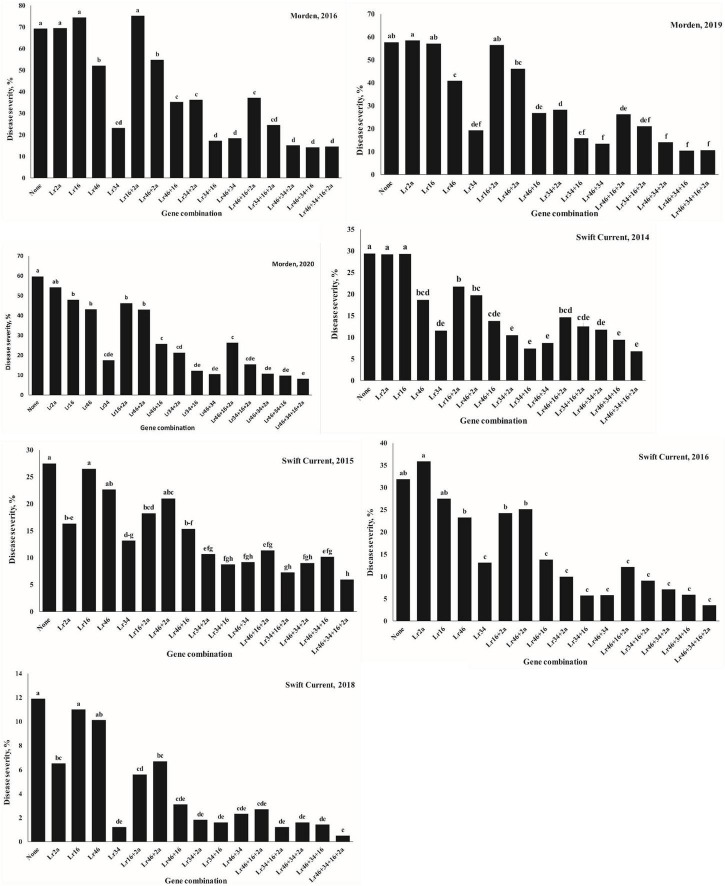
Effects of gene combinations on adult plant leaf rust response in the Carberry/Thatcher population evaluated near Morden, AB and Swift Current, SK in different years.

The ANOVA results for gene combination analysis using SAS aimed at investigating the effects of various gene combinations can be seen in Figure 4 and Supplementary Table 3. The results showed that the association of the *Lr34* gene with *Lr46*, *Lr16*, or *Lr2a* generally increased the level of disease resistance. At the Morden location in both 2019 and 2020, the effect of *Lr2a* or *Lr16* alone was marginal, whereas *Lr46* was moderately effective and *Lr34* was more effective (Figure 4). When *Lr46* and *Lr34* were combined in the same lines, these had a very good level of resistance, particularly when combined with *Lr2a* and/or *Lr16*.

## Discussion

The variation from moderate to high correlation values observed in the leaf rust severity was consistent with the plotted distributions of the CT population across environments and suggested a partial differential response to the environment. These results were suggestive of a leaf rust resistance complex of genes in Carberry, which is supported by low to moderate heritability values. Genetic analysis confirmed the complex nature of leaf rust resistance in Carberry that has been expressed at a high level since its commercial release with the identification of three seedling genes (*Lr2a*, *Lr16*, *Lr23*) and three APR genes (*Lr13*, *Lr34*, *Lr46*). We also detected minor QTL on chromosomes, 2B, 4A, 4B, 5A, and 5B that contributed to Carberry’s leaf rust resistance. The analysis of two-way epistatic effects revealed interactions between *Lr34* and *Lr46*, and *Lr34* and *Lr16*, which significantly boosted the resistance of Carberry.

The roughly bimodal distributions of the population observed at Morden 2016 and 2019, could be due to the high expression of the 7D QTL (*Lr34*) behaving like a qualitative gene explaining up to 42% of the phenotypic variation in 2016, and 38% in 2019, and low or insignificant expression by other Carberry genes/QTL. The first mode of the DH lines expressed a low-disease severity similar to the resistant parent, Carberry, whereas the other group had high-disease severity which was similar to the susceptible parent, Thatcher. Besides having a large effect by itself, *Lr34* also interacted with other genes forming two large groups, those with *Lr34* and those without. However, there were also DH lines that could not be categorized into Carberry type or Thatcher type due to the presence of the minor effect QTL which was segregating in the population. For example, besides the 7D QTL, three other QTL with a cumulative phenotypic effect of 18% segregated in 2016, and four QTL which cumulatively explained 25% of the phenotypic variation segregated in 2019 (Table 5).

Of the two resistance QTL detected using the field data on the chromosome arm 2BS, *QLr.spa-2B.1* corresponded with *Lr16.* The consistent expression of *Lr16* in most of the field tests in the current study was irrespective of the presence of races partially virulent on the gene in Canada (Samborski, 1984; McCallum et al., 2016, 2020). Predictive markers for *Lr16*, such as SSR markers, *wmc661, wmc764*, and *gwm210* were located nearby *QLr.spa-2B.1* in a high-density consensus map published by Bokore et al. (2020), while SNP marker, *BS00099465_51* mapped within the *QLr.spa-2B.1* QTL interval (Kassa et al., 2017; Figure 3). The presence of *Lr16* in Carberry was puzzling based on the gene determinations in the parental lines, Alsen and Superb, which did not detect *Lr16* in either of the parents, Superb (McCallum and Seto-Goh, 2010) and Alsen (Oelke and Kolmer, 2005). However, our seedling leaf rust assays and the use of predictive KASP markers supported the QTL analysis that Carberry carries *Lr16*. The puzzle was solved with the testing of multiple parental plants and although all the Superb plants tested were negative for *Lr16*, the Alsen plants tested were heterogeneous for *Lr16*. Thus, Carberry inherited *Lr16* from Alsen. Bokore et al. (2020) mapped the *Lr16* in Carberry using different mapping populations. Many other Canadian wheat varieties are known to have *Lr16* (McCartney et al., 2005; McCallum et al., 2016; Kassa et al., 2017; Toth et al., 2018).

Both of the parents of Carberry, Superb and Alsen, were reported to have the seedling leaf rust resistance gene, *Lr10* (Oelke and Kolmer, 2005; McCallum and Seto-Goh, 2010). However, no QTL was detected for *Lr10*, and seedling testing with two *Lr10* avirulent isolates indicated that the population did not segregate for *Lr10* and that Carberry did not have *Lr10*. When seed stocks of the parents that were used to make Carberry were tested, Superb appeared to be uniformly resistant to an *Lr10* avirulent isolate, but Alsen had a small proportion of susceptible plants demonstrating that these plants did not have *Lr10.* Therefore, it is likely that the Alsen plant that was crossed to produce Carberry had *Lr16* but lacked *Lr10*.

The detection of the third 2BS gene, *Lr23*, with the seedling test, but not in field trials, suggested that the ineffectiveness of the gene in the field was attributed to virulent races or a weak expressivity of the gene on adult plants. The frequency of virulence to *Lr23* varied from 37.5 to 56.1% from 2015–2019 in Canada (McCallum et al., 2021). Oelke and Kolmer (2005) reported one of Carberry’s parents, Alsen possesses *Lr23*. McIntosh and Dyck (1975) indicated the presence of an unknown gene in Thatcher that inhibits the expression of *Lr23* under Canadian field conditions, and partially in Australian conditions. Originally believed to be derived from a durum wheat cultivar Gaza, *Lr23* was later introgressed into hexaploid wheat (McIntosh and Dyck, 1975).

A third seedling gene identified in Carberry, *Lr2a* was determined by the seedling test and revealed by field data. Carberry was expected to carry *Lr2a* as both its parents, Alsen (Oelke and Kolmer, 2005) and Superb (McCallum and Seto-Goh, 2010) are reported to possess this gene. *Lr2a* is present in a wide range of North American wheat germplasm (Oelke and Kolmer, 2004; McCallum et al., 2016). *Lr2a* which corresponded with the field QTL, *QLr*.*spa-2D* showed a minor effect on DS and it was significant only in one environment. The effectiveness of the *Lr2a* gene in only one out of seven environments is consistent with the frequency of isolates virulent on the gene in the *P. triticina* population in western Canada dramatically keep increasing after the year 2000 (McCallum and Seto-Goh, 2010; McCallum et al., 2020). As the gene sometimes synergistically interacts with *Lr34*, it could still be valuable in resistance breeding.

Superb was reported not to have any APR genes (McCallum and Seto-Goh, 2010), and Alsen was reported to have *Lr13* and *Lr34* (Oelke and Kolmer, 2005). However, the current analysis demonstrated that *Lr46* was an important component of the resistance in Carberry. Carberry’s *QLr.spa-1B* QTL is the same as the slow rusting or APR and the pleiotropic gene, *Lr46*, located on the chromosome arm 1BL (Singh et al., 1998; William et al., 2003; Lillemo et al., 2013). The QTL at *QLr.spa-1B* was also effective against stem rust (data not shown). Studies are limited in showing the presence of *Lr46* in Canadian wheat germplasm. It was recently demonstrated that Superb has *Lr46* (Lewarne, 2021), which would have been the donor parent for *Lr46* in Carberry. Furthermore, recently published studies indicated a QTL on 1BL that was associated with multiple disease resistance in Carberry and Vesper that could be *Lr46* (Bokore et al., 2017, Bokore et al., 2020). The detection of the *QLr.spa-1B (Lr46)* in five out of seven environments with the current study indicates the importance of this gene in resistance breeding.

The *QLr.spa-7D* QTL on 7DS represented the major slow rusting pleiotropic gene *Lr34.* As opposed to *Lr46*, *Lr34* is common in many wheat varieties in Canada (McCallum and DePauw, 2008; McCallum et al., 2012, 2016) and several other countries (Singh et al., 2011; Lillemo et al., 2013). The presence of the *Lr34* gene in Carberry has been documented in various reports (McCallum et al., 2012; Randhawa et al., 2013; Bokore et al., 2020), and it was transferred to Carberry from Alsen (Oelke and Kolmer, 2005).

The QTL on chromosome 2B, *QLr.spa-2B.2*, corresponding with APR gene *Lr13*. The detection of this gene in Carberry was expected as one of its parents, Alsen, possesses the gene (Oelke and Kolmer, 2005). *Lr13* linked markers, *Excalibur_c26042_260* and *wsnp_Ku_c4042_7375890*, reported by Zhang et al. (2016), were placed within 5.15 and 8.58 cM distance from the *QLr.spa-2B.2* Carberry QTL peak marker, *Excalibur_c45094_602*, on the genetic map of the CT population (Figure 3). The gene is generally effective at the early postseedling stage (Dyck et al., 1966; Zhang et al., 2016). *Lr13* is a recessive gene as described by Dyck et al. (1966) in a study made using a cross of Thatcher/Manitou, the Canadian variety Manitou, possessing the gene, was originally transferred from Frontana in which it behaved partially dominant. This gene has been widely deployed in the Canadian and American wheat germplasm (McCallum et al., 2016), including Alsen, the immediate parent of Carberry’s immediate parent Alsen (Oelke and Kolmer, 2005). Zhang et al. (2016) reported that *Lr13* is the same gene as *Ne2m*, a gene known to govern hybrid necrosis in wheat. In indoor adult plant testing, we established the presence of *Lr13* in the progeny of the population of Carberry/Thatcher. Given that hybrids between Carberry and Kubanka (*Ne1* carrier) exhibited progressive necrosis, the presence of *Lr13* in Carberry was confirmed. The gene, *Lr13* is an example of an APR gene that has a race-specific response to leaf rust.

Carberry additionally has genes with relatively small effects on disease resistance identified on the chromosome arms, 4AS, 4BS, 5AL, and 5BS, which were significant in only one or a maximum of two out of seven environments. Despite the detection of several QTL in the resistant parent Carberry, we found that no QTL was contributed by the susceptible parent, Thatcher. The minor genes identified in Carberry may play a cumulative role in the resistance by acting in the synergy that is at a level that we could not detect in our epistasis analysis. Some of these minor QTLs were found to be close to genomic regions that are associated with other rust species on a hexaploid wheat consensus map (Bokore et al., 2020), making them useful regions to consider in resistance breeding. For example, the 4AS leaf rust QTL marker *RAC875_rep_c70416_332* was placed only at 0.25 cM proximal to *BobWhite_c20163_456*, a marker for stripe rust resistance similarly reported in Carberry (Bokore et al., 2020). Additionally, *BS00095286_51* associated with the 4BS leaf rust QTL was located at 31.5 cM proximal to *wmc617*, an SSR marker for resistance to stem rust race Ug99 in Carberry (Singh et al., 2013) and 37.2 cM proximal to *Tdurum_contig27799_114*, a marker for a stripe rust resistance QTL which was similarly reported in Carberry (Bokore et al., 2020).

Understanding the epistatic genetic effects of multiple leaf rust resistance genes is useful to develop wheat varieties with appropriate gene combinations and durable resistance. Results of the present study demonstrated that the strength of Carberry’s years of leaf rust resistance in the farmer’s fields in Canada may, in part, be attributable to the synergistic additive by additive epistatic effects of *Lr34* with *Lr46, Lr16*, and/or *Lr2a*. For over 100 years, *Lr34* has remained effective against the leaf rust pathogen. *Lr34* has been cloned and encoded an ABC transporter protein unlike cloned all stage resistance genes, but how this protein confers resistance to rust pathogen is not known (Krattinger et al., 2009). Singh et al. (2011) indicated that gene combinations could minimize the development of virulent races on race-specific resistance genes. Also, wheat varieties having *Lr34* in combination with other genes are more durable compared with varieties that lack *Lr34* (Oelke and Kolmer, 2004; Singh et al., 2006, 2011). Singh et al. (2011) reported that combinations of 4 to 5 APR genes usually result in “near immunity” or high level resistance.

In conclusion, since its registration in Canada in 2009, the hexaploid spring wheat cultivar, Carberry, was grown over an extensive area in Canada for several years and maintained a high level of leaf rust (*Puccinia triticina* Eriks.) resistance. The present study characterized the genetic basis of what has turned out to be durable resistance in Carberry through several race-specific and non-specific race-resistance genes. Using the adult plant leaf rust response data, we identified nine QTLs located on chromosomes 1B, 2B.1, 2B.2, 2D, 4A, 4B, 5A, 5B, and 7D some of which represented previously documented genes. For example, the resistance on 1B corresponded with *Lr46*, 7D with *Lr34*, one of the QTL on 2B with *Lr13*, the other QTL on 2B with *Lr16*, and 2D with *Lr2a*. In addition to field studies, our seedling tests revealed race-specific genes, *Lr2a*, *Lr16*, and *Lr23*. Although *Lr2a* and *Lr16* were also revealed using adult plant response data, *Lr23* did not show any significant effect in adult plants due to the presence of gene-specific virulent races in recent years. Synergistic epistatic effects were revealed for *Lr34* with *Lr46*, *Lr16*, or *Lr2a* with a combination of these genes contributing to higher resistance. Single events of negative interactions were detected between *Lr16* and *Lr46*, and between *Lr2a* and an unknown QTL on chromosome 2B. However, these gene combinations had reduced the disease symptoms compared to each gene alone. Generally, the durability of leaf rust resistance in Carberry could be attributed to *Lr34* and *Lr46*, because the resistance has remained effective even though the *P. triticina* population has evolved virulence to *Lr2a, Lr13, Lr16*, and *Lr23*.

## Data Availability Statement

The original contributions presented in the study are included in the article/Supplementary Material, further inquiries can be directed to the corresponding author/s.

## Author Contributions

RK, RC, and RD developed the mapping population. RK, RC, BDM, CH, and FB conceived, designed, and supervised the study. BDM, FB, RK, and RC performed the field trials and phenotyping. BDM performed seedling resistance trials. CH performed the hybrid necrosis test and predictive marker tests. FB, BM, CP, and AN’D performed genotyping, genetic data mining, and genetic mapping. FB analyzed the data and drafted the manuscript. FB, BDM, RK, CH, RC, and RD revised the manuscript. All authors reviewed the manuscript.

## Conflict of Interest

The authors declare that the research was conducted in the absence of any commercial or financial relationships that could be construed as a potential conflict of interest.

## Publisher’s Note

All claims expressed in this article are solely those of the authors and do not necessarily represent those of their affiliated organizations, or those of the publisher, the editors and the reviewers. Any product that may be evaluated in this article, or claim that may be made by its manufacturer, is not guaranteed or endorsed by the publisher.

## Footnotes

1
https://www.grainscanada.gc.ca/en/grain-research/statistics/varieties-by-acreage/
