# Supplementary material for: A Combination of Leaf Rust Resistance Genes, Including Lr34 and Lr46, Is the Key to the Durable Resistance of the Canadian Wheat Cultivar, Carberry
Source: Front Plant Sci. 2022 Jan 6;12:775383. doi: 10.3389/fpls.2021.775383 (PMC8770329; doi:10.3389/fpls.2021.775383)
Supplement: Supplementary file 1 [file Table_1.docx]

**SUPPLEMENTAL TABLE 1 |** Determination of the leaf rust resistance gene *Lr16* in Superb and Alsen, the parental lines for Carberry.

|  | **Puccinia triticina race** | **KASP marker** | |
| --- | --- | --- | --- |
| **Line Identity** | **20-140-1 TBRD** | **kwm677** | **kwm849** |
| BW252 (Superb) | 1 1+ 2- | B | B |
| BW252 (Superb) | 1 1+ 2- | B | B |
| BW252 (Superb) | 1 1+ 2- | B | B |
| BW252 (Superb) | 1 1+ 2- | B | B |
| BW252 (Superb) | 1 1+ 2- | B | B |
| BW252 (Superb) | 1 1+ 2- | B | B |
| BW252 (Superb) | 1 1+ 2- | B | B |
| BW252 (Superb) | 1 1+ 2- | B | B |
| BW252 (Superb) | 1 1+ 2- | B | B |
| BW252 (Superb) | 1 1+ 2- | B | B |
| BW252 (Superb) | 1 1+ 2- | B | B |
| BW252 (Superb) | 1 1+ 2- | B | B |
| BW252 (Superb) | 1 1+ 2- | B | B |
| BW252 (Superb) | 1 1+ 2- | B | B |
| BW252 (Superb) | 1 1+ 2- | B | B |
| BW252 (Superb) | 1 1+ 2- | B | B |
| Thatcher | 3+ | B | B |
| Lr2a | 3+ | B | B |
| Lr10 | 2- | B | B |
| Lr23 | 3 | B | B |
| Lr16 | 1+ | A | A |
| Carberry | ;1- | A | A |
| Thatcher | 3+ | B | B |
| Lr2a | 3+ | B | B |
| Lr10 | 2- | B | B |
| Lr23 | 2 3 | B | B |
| Lr16 | 11- | A | A |
| Carberry | ;1- | A | A |
| Alsen (ND716) | ;1= | B | B |
| Alsen (ND716) | ; | A | A |
| Alsen (ND716) | ;1= | B | B |
| Alsen (ND716) | 1- | A | A |
| Alsen (ND716) | ;1= | B | B |
| Alsen (ND716) | ;1= | B | B |
| Alsen (ND716) | ;1= | A | A |
| Alsen (ND716) | 3+ | B | B |
| Alsen (ND716) | ;1- | A | A |
| Alsen (ND716) | ; | A | A |
| Alsen (ND716) | ;1- | A | A |
| Alsen (ND716) | ;1= | B | B |
| Alsen (ND716) | ;1= | A | A |
| Alsen (ND716) | ;1- | A | A |
| Alsen (ND716) | 3+ | B | B |
| Alsen (ND716) | ;1= | A | A |
|  | **Puccinia triticina race** | **KASP marker** | |
| **Line Identity** | **19-123-2 TBGJ** | **kwm677** | **kwm849** |
| BW252 (Superb) | 3 | B | B |
| BW252 (Superb) | 3 | B | B |
| BW252 (Superb) | 3 3+ | B | B |
| BW252 (Superb) | 3 3+ | B | B |
| BW252 (Superb) | 3 | B | B |
| BW252 (Superb) | 3 | B | B |
| BW252 (Superb) | 3 3+ | B | B |
| BW252 (Superb) | 3 3+ | B | B |
| BW252 (Superb) | 3 | B | B |
| BW252 (Superb) | 3 | B | B |
| BW252 (Superb) | 3- | B | B |
| BW252 (Superb) | 3- | B | B |
| BW252 (Superb) | 3- | B | B |
| BW252 (Superb) | 3 | B | B |
| BW252 (Superb) | 3 | B | B |
| BW252 (Superb) | 3 3+ | B | B |
| Thatcher | 3+ | B | B |
| Lr2a | 3+ | B | B |
| Lr10 | 3 | B | B |
| Lr23 | 3- | B | B |
| Lr16 | ;1-- | A | A |
| Carberry | ;1-- | A | A |
| Thatcher | 3+ | B | B |
| Lr2a | 3+ | B | B |
| Lr10 | 3 | B | B |
| Lr23 | 3- | B | B |
| Lr16 | ;1-- | A | A |
| Carberry | ; | A | A |
| Alsen (ND716) | 3+ | B | B |
| Alsen (ND716) | ; | A | A |
| Alsen (ND716) | 3- | B | B |
| Alsen (ND716) | 3+ | B | B |
| Alsen (ND716) | ; | A | A |
| Alsen (ND716) | ; | A | A |
| Alsen (ND716) | ;1-- | A | A |
| Alsen (ND716) | 3 | B | B |
| Alsen (ND716) | 3+ | B | B |
| Alsen (ND716) | 3- | B | B |
| Alsen (ND716) | 1- | A | A |
| Alsen (ND716) | ; | A | A |
| Alsen (ND716) | 1 1+ | A | A |
| Alsen (ND716) | ;1-- | A | A |
| Alsen (ND716) | 3- | B | B |
| Alsen (ND716) | ;1-- | A | A |
