# Supplementary material for: A Combination of Leaf Rust Resistance Genes, Including Lr34 and Lr46, Is the Key to the Durable Resistance of the Canadian Wheat Cultivar, Carberry
Source: Front Plant Sci. 2022 Jan 6;12:775383. doi: 10.3389/fpls.2021.775383 (PMC8770329; doi:10.3389/fpls.2021.775383)
Supplement: Supplementary file 2 [file Table_2.docx]

**SUPPLEMENTAL TABLE 2 |** Summary statistics for the Carberry/Thatcher population high density SNP linkage map.

| **Chromosome** | **Number of markers** | **Length, cM** | **Density, cM/marker** | **Biggest interval, cM** |
| --- | --- | --- | --- | --- |
| 1A | 572 | 238.0 | 0.42 | 10.8 |
| 1B.1 | 390 | 142.9 | 0.37 | 22.9 |
| 1B.2 | 92 | 16.1 | 0.17 | 2.1 |
| 1D | 76 | 74.5 | 0.98 | 20.3 |
| 2A | 376 | 190.4 | 0.51 | 22.6 |
| 2B | 1300 | 357.1 | 0.27 | 27.7 |
| 2D | 38 | 93.6 | 2.46 | 23.1 |
| 3A | 474 | 243.4 | 0.51 | 23.9 |
| 3B.1 | 107 | 110.4 | 1.03 | 28.0 |
| 3B.2 | 58 | 24.0 | 0.41 | 2.8 |
| 3D.1 | 136 | 135.5 | 1.00 | 21.3 |
| 3D.2 | 109 | 5.5 | 0.05 | 1.7 |
| 4A.1 | 194 | 79.6 | 0.41 | 8.0 |
| 4A.2 | 228 | 25.1 | 0.11 | 10.3 |
| 4B | 403 | 172.4 | 0.43 | 14.0 |
| 5A | 485 | 253.8 | 0.52 | 20.7 |
| 5B | 831 | 323.6 | 0.39 | 21.4 |
| 5D.1 | 129 | 44.8 | 0.35 | 13.2 |
| 5D.2 | 31 | 15.0 | 0.48 | 4.8 |
| 5D.3 | 5 | 6.4 | 1.74 | 5.2 |
| 6A | 301 | 180.1 | 0.60 | 25.7 |
| 6B | 689 | 272.6 | 0.40 | 37.1 |
| 6D.1 | 84 | 32.4 | 0.39 | 11.5 |
| 6D.2 | 43 | 8.3 | 0.19 | 2.7 |
| 7A | 678 | 279.0 | 0.41 | 23.5 |
| 7B.1 | 355 | 128.2 | 0.36 | 11.2 |
| 7B.2 | 106 | 70.6 | 0.67 | 15.9 |
| 7D | 70 | 122.9 | 1.76 | 25.3 |
| Total | 8360 | 3645.8 | 0.44 |  |
