# Supplementary material for: A Combination of Leaf Rust Resistance Genes, Including Lr34 and Lr46, Is the Key to the Durable Resistance of the Canadian Wheat Cultivar, Carberry
Source: Front Plant Sci. 2022 Jan 6;12:775383. doi: 10.3389/fpls.2021.775383 (PMC8770329; doi:10.3389/fpls.2021.775383)
Supplement: Supplementary file 3 [file Table_3.docx]

**SUPPLEMENTAL TABLE 3 |** Mean comparison by Duncan’s Multiple Range Test of leaf rust severity and infection response for gene interactions between four QTL alleles/genes on chromosomes 1BL (*Lr46*) associated with SNP marker *BS00000010_51*, 7DS (*Lr34*) with *Kukri_c32845_116*, *Lr16* (2B) with *BS00099465_51*, and 2DS (*Lr2a*) with *wsnp_Ex_c6400_11123059.*

| **Gene or gene combination** | **Number of positive loci** | **Morden 2020** | **Morden 2019** | **Morden 2016** | **Swift Current 2018** | **Swift Current 2016** | **Swift Current 2015** | **Swift Current 2014** |
| --- | --- | --- | --- | --- | --- | --- | --- | --- |
| Disease severity,% | | | | | | | | |
| None | 0 | 59.8a* | 57.7ab | 69.3a | 11.9a | 31.9ab | 27.5a | 29.4a |
| *Lr2a* | 1 | 54.2ab | 58.4a | 69.4a | 6.5bc | 35.9a | 16.3b-e | 29.2a |
| *Lr16* | 1 | 48.0b | 57.1ab | 74.5a | 11.0a | 27.5ab | 26.5a | 29.3a |
| *Lr46* | 1 | 43.2b | 40.8c | 52.1b | 10.1ab | 23.3b | 22.6ab | 18.7bcd |
| *Lr34* | 1 | 17.6cde | 19.2def | 23.1cd | 1.2de | 13.1c | 13.1d-g | 11.6de |
| *Lr16/Lr2a* | 2 | 46.3b | 56.4ab | 75.2a | 5.6cd | 24.3b | 18.2bcd | 21.8b |
| *Lr46/Lr2a* | 2 | 43.1b | 46.1cb | 54.8b | 6.7bc | 25.13b | 21abc | 19.7bc |
| *Lr46/Lr16* | 2 | 25.8c | 26.8de | 35.1c | 3.1cde | 13.8c | 15.3b-f | 13.8cde |
| *Lr34/Lr2a* | 2 | 21.4cd | 28.1d | 36.2c | 1.8de | 10.0c | 10.6efg | 10.5e |
| *Lr34/Lr16* | 2 | 12.3de | 15.7ef | 17.2d | 1.6de | 5.7c | 8.7fgh | 7.4e |
| *Lr46+Lr34* | 2 | 10.5de | 13.4f | 18.4d | 2.3cde | 5.8c | 9.1fgh | 8.7e |
| *Lr46/Lr16/Lr2a* | 3 | 26.3c | 26.2de | 37.1c | 2.7cde | 12.1c | 11.3efg | 14.6bcd |
| *Lr34/Lr16/Lr2a* | 3 | 15.5cde | 21.0def | 24.5cd | 1.2de | 9.1c | 7.2gh | 12.5cde |
| *Lr46/Lr34/Lr2a* | 3 | 10.7de | 13.9f | 15.0d | 1.6de | 7.1c | 9.0fgh | 11.8de |
| *Lr46/Lr34/Lr16* | 3 | 9.8de | 10.3f | 14.2d | 1.4de | 5.9c | 10.1efg | 9.4e |
| *Lr46/Lr34/Lr16/Lr2a* | 4 | 8.2e | 10.6f | 14.5d | 0.5e | 3.5c | 5.9h | 6.8e |
| S.E.(P < 5%) | | 14.6 | 15.5 | 19.1 | 5.5 | 12.5 | 8.6 | 9.8 |
| **Infection response** | | | | | | | | |
| None | 0 | 6.6a | 6.6a | 7.9a | 4.5bcd | 5.9ab | 6.5ab | 6.9a |
| *Lr2a* | 1 | 6.3ab | 6.7a | 7.4a | 5.5a | 6.6a | 6.4ab | 6.5a |
| *Lr16* | 1 | 5.8abc | 6.2ab | 8.0a | 4.8abc | 4.3de | 5.7a-d | 6.9a |
| *Lr46* | 1 | 5.4bcd | 5.5bc | 6.3b | 5.0ab | 4.7cd | 6.3abc | 6.4ab |
| *Lr34* | 1 | 4.6def | 4.1e | 3.9e | 3.9c-f | 4.2de | 5.1efg | 4.7cde |
| *Lr16/Lr2a* | 2 | 5.7abc | 6.1ab | 7.9a | 4.8abc | 4.7cd | 5.8a-d | 6.6a |
| *Lr46/Lr2a* | 2 | 5.6bc | 5.8abc | 6.4b | 5.1ab | 5.4cb | 6.7a | 6.3ab |
| *Lr46/Lr16* | 2 | 5.0cde | 5.0cd | 5.1dc | 4.2b-f | 4.0de | 5.1efg | 5.2cde |
| *Lr34/Lr2a* | 2 | 4.3efg | 5.1cd | 4.7dce | 4.3b-e | 4.4de | 5.1efg | 4.5de |
| *Lr34/Lr16* | 2 | 4.2efg | 4.3de | 3.9e | 3.3f | 3.8de | 4.2fg | 4.3e |
| *Lr46+Lr34* | 2 | 3.7fg | 4.1de | 3.8e | 3.6def | 3.9de | 4.9efg | 4.9cde |
| *Lr46/Lr16/Lr2a* | 3 | 5.0cde | 5.0cd | 5.6bc | 4.6a-d | 4.1de | 5.3def | 5.3cd |
| *Lr34/Lr16/Lr2a* | 3 | 4.6def | 4.3de | 4.5de | 4.2b-f | 3.9de | 4.5efg | 4.7cde |
| *Lr46/Lr34/Lr2a* | 3 | 4.6def | 4.5de | 4.1de | 4.4bcd | 3.8de | 5.4b-e | 5.5bc |
| *Lr46/Lr34/Lr16* | 3 | 4.0efg | 3.7e | 3.7e | 3.3f | 3.9de | 4.0g | 4.6cde |
| *Lr46/Lr34/Lr16/Lr2a* | 4 | 3.6g | 3.7e | 3.7e | 3.4ef | 3.5e | 4.3efg | 4.4de |
| S.E. (P < 5%) |  | 1.2 | 1.2 | 1.4 | 1.2 | 1.3 | 1.4 | 1.2 |

*Means with the same letter in a column are not significantly different at 5% probability level.
